# Bursting the Bubble: Spontaneous Pneumothorax Secondary to Pneumocystis jirovecii Pseudocysts in a Young Diabetic Patient

**DOI:** 10.7759/cureus.95813

**Published:** 2025-10-31

**Authors:** Salma Abrar, Hanane Ikrou, Ghita Baani, Halloumi Oussama, Hind Serhane

**Affiliations:** 1 Pulmonology Department, Hassan II Regional Hospital, Souss-Massa University Hospital, LARISS Laboratory, Faculty of Medicine and Pharmacy, Ibn Zohr University, Agadir, MAR; 2 Critical Care Medicine Department, Souss Massa University Hospital, Agadir, MAR; 3 Pulmonary Medicine Department, Souss Massa University Hospital, Agadir, MAR; 4 Pulmonology Department, Souss Massa University Hospital, Faculty of Medicine and Pharmacy, Ibn Zohr University, Agadir, MAR; 5 Pulmonology Department, Centre Hospitalo-Universitaire Souss Massa, Agadir, MAR

**Keywords:** non-hiv immunosuppression, pneumocystis jirovecii pneumonia, pulmonary pseudocysts, spontaneous pneumothorax, type 1 diabetes mellitus

## Abstract

*Pneumocystis jirovecii* pneumonia (PJP) is a well-known opportunistic infection in HIV-infected patients, but remains rare and often under-recognized in type 1 diabetic (T1D) patients. We report the case of a young woman with poorly controlled T1D, HIV-seronegative, who developed severe PJP complicated by spontaneous pneumothorax and reversible pulmonary pseudocysts. The diagnosis was confirmed by PCR on induced sputum. The patient received treatment with trimethoprim-sulfamethoxazole, corticosteroids, and thoracic drainage for pneumothorax management, resulting in progressive clinical improvement. This atypical clinical presentation illustrates the diagnostic and therapeutic challenges in this at-risk population. This case highlights the importance of suspecting PJP in T1D patients presenting with acute respiratory failure, even in the absence of classical immunosuppressive factors, and underscores the need for close monitoring of cystic complications to prevent potentially severe pneumothoraces.

## Introduction

*Pneumocystis jirovecii* pneumonia (PJP) is a major opportunistic infection primarily affecting immunocompromised patients, especially those with HIV/AIDS [1]. However, the epidemiology of PJP is evolving, with an increasing number of cases reported in non-HIV patients presenting other forms of immunosuppression, such as individuals with diabetes mellitus, autoimmune diseases, or those receiving immunosuppressive therapies [2]. Type 1 diabetes (T1D), characterized by autoimmune destruction of pancreatic β-cells leading to absolute insulin deficiency, induces profound immune dysregulation, including impaired neutrophil function and T-lymphocyte responses, which increases susceptibility to opportunistic infections [3].

In diabetic patients, chronic hyperglycemia exacerbates these immune deficits, promoting the development of severe infections, including PJP, which may present with atypical clinical and radiological features such as pulmonary cystic lesions and spontaneous pneumothorax [4]. These complications, more frequent in non-HIV patients, complicate diagnosis and management, requiring heightened clinical vigilance. Diagnosis relies on molecular detection of *Pneumocystis jirovecii* by PCR on respiratory samples, particularly valuable in non-HIV patients where fungal burden is often low. Standard treatment combines trimethoprim-sulfamethoxazole (TMP-SMX) and corticosteroids in cases of severe hypoxemia, with careful monitoring for mechanical complications [5].

We report the case of a young woman with poorly controlled T1D, HIV-seronegative, who developed severe PJP complicated by spontaneous pneumothorax and reversible pulmonary pseudocysts, illustrating the diagnostic and therapeutic challenges in this population.

## Case presentation

We report the case of a 20-year-old female agricultural worker with a four-year history of poorly controlled type 1 diabetes mellitus (HbA1c 12.8%) due to poor treatment adherence, with no prior history of chronic respiratory or chest disease. She presented to the emergency department with acute severe dyspnea (Modified Medical Research Council (mMRC) grade 4), fever, and productive cough with greenish sputum. Notably, she had discontinued her insulin therapy five days prior, precipitating diabetic ketoacidosis.

On admission, clinical examination revealed oxygen saturation (SpO₂) of 70% on room air, improving to 94% with a non-rebreathing mask delivering 15 L/minute of supplemental oxygen. Blood pressure was 80/70 mmHg, heart rate 150 beats per minute, temperature 38.5°C, and respiratory rate 30 breaths per minute with suprasternal and intercostal retractions. Pulmonary auscultation revealed bilateral basal crackles and signs of consolidation on percussion.

Laboratory investigations at admission and post-treatment are summarized in Table 1.

**Table 1:** Summary of biological parameters.

Parameter	Initial Value	Post-treatment Value	Reference Values
Capillary blood glucose (g/L)	3.0	1.2	0.7 to 1.1 g/L
HbA1c (%)	12.8	7.5	< 6.0% for well-controlled diabetes
Leukocytes (/mm³)	15,975	<10,000	4,000 to 10,000 /mm³
C-reactive protein (mg/L)	242	6.23	< 5 mg/L
PaO₂ (mmHg) on room air	42.4	>80	75 to 100 mmHg
HIV serology	Negative	-	Negative
Serum protein electrophoresis	Normal	-	Normal
*Pneumocystis jirovecii* PCR on induced sputum	Positive	Negative	Negative
Microscopic identification of *Pneumocystis jirovecii* (special stains)	Positive result obtained after initiation of treatment (performed after PCR positivity )	-	Positive confirms diagnosis
Sputum bacterial smear and culture	No pathogenic growth	-	Negative
Sputum smear for acid-fast bacilli (AFB)	Negative	-	Negative
GeneXpert MTB/RIF on sputum	Negative	-	Negative
Methicillin-resistant *Staphylococcus aureus* (MRSA) screening	Negative	-	Negative

During outpatient follow-up visits at one and three months, the patient demonstrated sustained clinical stability without any infectious or respiratory relapse. Importantly, her glycemic control improved significantly, with HbA1c levels decreasing from 12.8% to 7.5% under an optimized intensive insulin regimen, reflecting effective metabolic management that likely contributed to her favorable respiratory recovery.

**Figure 1:**
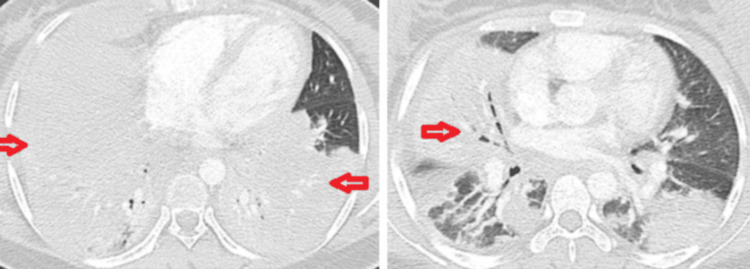
Chest CT at admission. Well-defined areas of consolidation in the bilateral lower lobes, the right middle lobe, and the lingular segment, with air bronchograms and bilateral peribronchovascular thickening. A mosaic perfusion pattern is also noted, with alternating areas of hyper and hypoattenuation.

Chest computed tomography (CT) performed on admission demonstrated well-defined areas of consolidation in the bilateral lower lobes, right middle lobe, and lingula, with air bronchograms and bilateral peribronchovascular thickening. A bilateral pleural effusion was noted, more prominent on the right side. A mosaic perfusion pattern was observed, characterized by alternating areas of hyperattenuation and hypoattenuation (Figure 1).

The patient received comprehensive management, which included targeted antibiotic therapy initiated on hospital day 2 after obtaining a positive PCR result for *Pneumocystis jirovecii*. TMP-SMX was administered at a dose of 15 mg/kg/day for 21 days. Prednisolone was initiated in response to severe hypoxemia and administered over 21 days with tapering doses, according to current treatment guidelines: 40 mg twice daily for the first five days, 40 mg once daily for the next five days, and 20 mg once daily for the remaining 11 days. High-flow oxygen therapy was delivered via a non-rebreathing mask, targeting an SpO₂ above 95%. Intensive insulin therapy with insulin glargine (long-acting) and human insulin (short-acting) was employed to correct ketoacidosis and optimize glycemic control. The patient also received intravenous hydration and antipyretics to support recovery.

**Figure 2:**
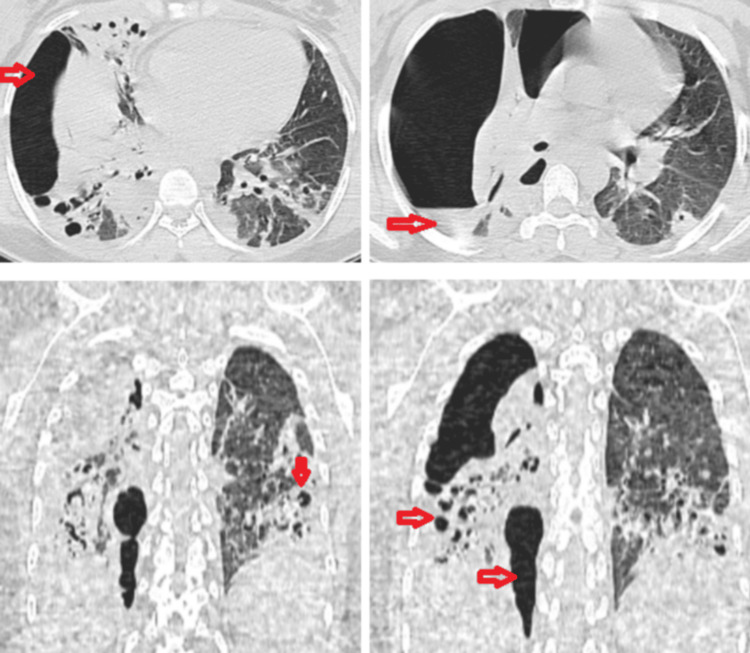
Chest CT on day 5 of hospitalization. Large right-sided pneumothorax with significant right lung atelectasis, associated with volume loss and architectural distortion. Presence of cylindrical bronchiectasis and bronchiolectasis in the right lung field, with marked peribronchial and peribronchiolar thickening. A cystic bronchiectasis is visible in the right posterior-basal segment, developing over an area of consolidation. Additional bronchiectases, mostly cylindrical, are located preferentially in the Fowler and posterior-basal segments of the lingular lobe, with signs of superinfection (peribronchial and peribronchiolar thickening) on a background of scattered consolidations. Persistent bilateral pleural effusion, more prominent on the right, along with bilateral peribronchovascular thickening.

Following initial improvement, on hospital day 5, the patient developed acute right-sided pleuritic chest pain described as stabbing, accompanied by severe dyspnea (mMRC grade 3). Physical examination revealed tachypnea at 30 breaths per minute, decreased oxygen saturation, and signs of respiratory distress, with clinical findings consistent with a right-sided pneumothorax. A chest CT scan confirmed a large right-sided pneumothorax with significant lung collapse and bronchiectatic changes (Figure 2).

A chest tube was inserted, resulting in complete re-expansion of the right lung without surgical intervention. From day 16 onward, the patient exhibited significant respiratory improvement, with SpO₂ reaching 90% on room air, resolution of fever and dyspnea, and normalization of inflammatory markers (CRP <10 mg/L, leukocytes <10,000/mm³).

**Figure 3:**
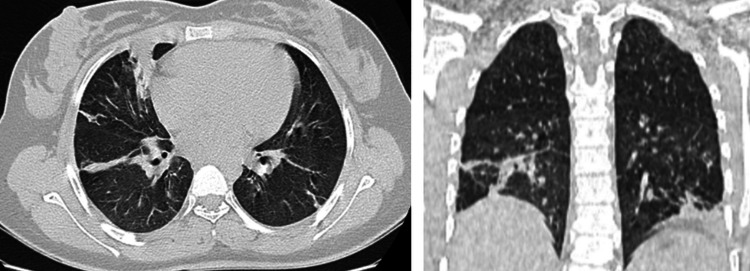
Chest CT one month later. Presence of reticulations and thickening of interlobular septa. Residual left basal consolidation and a perihilar consolidation on the right. Complete resolution of bronchial dilatation, suggesting regression of pseudocysts associated with *Pneumocystis jirovecii*.

Two weeks post-discharge (day 30), follow-up chest CT (Figure 3) demonstrated complete resolution of the previously observed bronchiectasis and pseudocysts, confirming their reversible nature. Residual findings were limited to minimal reticulations and septal thickening consistent with mild post-inflammatory fibrosis.

During outpatient follow-up visits at one and three months, the patient demonstrated sustained clinical stability without any infectious or respiratory relapse. Importantly, her glycemic control improved significantly, with HbA1c levels decreasing from 12.8% to 7.5% under an optimized intensive insulin regimen, reflecting effective metabolic management that likely contributed to her favorable respiratory recovery.

## Discussion

PJP is a well-known opportunistic infection in HIV-positive patients, but it is also observed in non-HIV individuals with functional immunosuppression, particularly in poorly controlled type 1 diabetic (T1D) patients. Diabetes induces a complex immune dysfunction, including impaired neutrophil chemotaxis and phagocytosis, as well as deficient T-cell responses, which promote the development of severe opportunistic infections such as PJP [2].

*Pneumocystis jirovecii* is an atypical human fungus transmitted via airborne routes. Following inhalation, it colonizes the pulmonary alveoli, where it multiplies and induces diffuse interstitial inflammation. This inflammation leads to thickening of the interalveolar septa and lymphocytic infiltration, impairing gas exchange and resulting in severe hypoxemia [6]. In the setting of immunosuppression, uncontrolled fungal proliferation can lead to alveolar necrosis and the formation of cystic or pseudocystic lesions, weakening the lung parenchyma and increasing the risk of spontaneous pneumothorax [7,8].

Typical radiographic features of PJP include bilateral ground-glass opacities. However, 20-30% of patients may have a normal chest X-ray. Chest CT is more sensitive and often reveals ground-glass opacities with septal thickening, consolidations, and cystic lesions predominantly located at the lung apices. These cystic lesions correspond to pseudocysts, which are transient bronchial dilatations resulting from alveolar destruction due to inflammation and necrosis. Several studies have reported that these pseudocysts may regress or resolve following effective treatment of PJP, reflecting their reversible nature linked to resolution of inflammation and tissue repair [9]. This reversibility was observed in our case, where cystic lesions disappeared during post-therapeutic follow-up, confirming that these pseudocysts are not permanent fibrotic changes but rather transient inflammatory phenomena.

However, the presence of such pseudocysts exposes patients to serious mechanical complications, especially spontaneous pneumothorax, which requires urgent management, often with chest tube drainage. Close monitoring of these lesions is therefore essential [10].

Diagnosis of PJP in non-HIV patients is often delayed due to a lower fungal burden, making microscopic detection less sensitive. PCR testing on respiratory samples, particularly induced sputum or bronchoalveolar lavage, has become the gold standard for rapid and reliable detection of *Pneumocystis jirovecii*. Early diagnosis is crucial to initiate appropriate therapy [11].

Treatment is based on a 21-day course of TMP-SMX, combined with corticosteroids in cases of severe hypoxemia to reduce pulmonary inflammation. Our patient received this standard therapy, along with chest drainage for management of the spontaneous pneumothorax, which led to a favorable outcome [12].

Current guidelines do not recommend routine prophylaxis in diabetic patients, but it may be considered in individuals with poor glycemic control (HbA1c >10%), recurrent opportunistic infections, or associated immunosuppression. Strict glycemic control is a key factor in reducing infection risk [13].

Learning points

PJP can occur even in young, HIV-negative patients with poorly controlled type 1 diabetes mellitus or other subtle immune dysfunctions.

These infections are often underrecognized and underestimated in non-HIV patients, leading to delayed diagnosis and poorer outcomes.

PCR provides a rapid and sensitive diagnostic tool that supports early therapeutic decisions, although microscopic identification using special stains remains the gold standard.

Radiological interpretation can be challenging; complications, such as pulmonary pseudocysts and spontaneous pneumothorax, may occur, the latter often secondary to cyst rupture, even after treatment initiation.

Clinicians should maintain a high index of suspicion for these complications, and close radiological and clinical monitoring is essential throughout the recovery phase.

Early recognition and multidisciplinary management involving infectious disease, pulmonology, and radiology specialists are crucial to prevent life-threatening complications.

With timely diagnosis and appropriate therapy, both clinical and radiological improvements can be remarkable, even in severe presentations.

## Conclusions

Severe PJP can occur, although extremely rarely, in HIV-seronegative patients with poorly controlled type 1 diabetes mellitus. This case illustrates that even in the absence of classical immunosuppressive factors, significant immune dysregulation related to chronic hyperglycemia may predispose to opportunistic infections. The patient developed transient pulmonary pseudocysts and spontaneous pneumothorax, which resolved with timely drainage and standard therapy. Early molecular confirmation on induced sputum, prompt initiation of TMP-SMX and corticosteroids for hypoxemia, and close imaging surveillance enabled full recovery. This report underscores the importance of maintaining a high index of suspicion for PJP in diabetic patients presenting with acute respiratory failure and of monitoring for delayed mechanical complications to prevent morbidity.
